# Maternal weight and birth outcomes among women on antiretroviral treatment from conception in a birth surveillance study in Botswana

**DOI:** 10.1002/jia2.25763

**Published:** 2021-06-27

**Authors:** Rebecca Zash, Ellen C Caniglia, Modiegi Diseko, Gloria Mayondi, Judith Mabuta, Rebecca Luckett, G Justus Hofmeyr, Chelsea Morroni, Doreen Ramogola‐Masire, Paige L Williams, Chloe Zera, Blair J Wylie, Joseph Makhema, Shahin Lockman, Roger L Shapiro

**Affiliations:** ^1^ Beth Israel Deaconess Medical Center Boston MA USA; ^2^ Botswana‐Harvard AIDS Institute Partnership Gaborone Botswana; ^3^ Harvard T.H. Chan School of Public Health Boston MA USA; ^4^ New York University School of Medicine New York NY USA; ^5^ University of Botswana Gaborone Botswana; ^6^ Liverpool School of Tropical Medicine Liverpool UK; ^7^ Brigham and Women’s Hospital Boston MA USA

**Keywords:** HIV in pregnancy, stillbirth, gestational weight gain, dolutegravir, efavirenz, medication exposure in pregnancy

## Abstract

**Introduction:**

Antiretrovirals such as dolutegravir (DTG) and tenofovir alafenamide (TAF) have been associated with excessive weight gain. The objective of this study was to understand the potential impact of ART‐associated weight gain on pregnancy outcomes among women living with HIV.

**Methods:**

Using data from the Tsepamo birth outcomes surveillance study in Botswana, we evaluated the relationship between maternal weight (and weight gain) and severe birth outcomes (very preterm delivery <32 weeks, very small for gestational age (SGA) <3rd percentile, perinatal death), macrosomia (birthweight > 4000 g) and maternal hypertension. We estimated the relative risk of each outcome by baseline weight (first weight in pregnancy <24 weeks) and second trimester average weekly weight gain (kg/week from 12 ± 2 to 24 ± 2 weeks) using log binomial regression and evaluated effect modification by ART regimen (DTG vs. Efavirenz (EFV)).

**Results:**

Of 22,828 women on ART at conception with singleton deliveries between August 2014 and April 2020, 16,300 (71.4%) had a weight measured <24 weeks’ gestation (baseline weight) and 4437 (19.2%) had weight measured both at 12 (±2) weeks and 24 (±2) weeks, allowing second trimester weight gain calculation. Compared to women with baseline weight 60 to 70 kg, low baseline weight (<50 kg) was associated with increased risk of very preterm delivery (aRR 1.30, 95% CI 1.03, 1.65) and very SGA (aRR1.96, 95% CI 1.69, 2.28). High baseline weight (>90 kg) was associated with increased risk of macrosomia (aRR 3.24, 95% CI 2.36, 4.44) and maternal hypertension (aRR 1.79, 95% CI 1.62, 1.97). Baseline weight was not associated with stillbirth or early neonatal death. For all outcomes, second trimester weight gain showed weaker associations than did baseline weight. Duration of pre‐pregnancy ART (years) was associated with higher baseline weight for DTG but not for EFV, and the risk of maternal hypertension by baseline weight category was higher for DTG than EFV for all strata.

**Conclusions:**

ART regimens associated with weight gain may reduce the number of women at risk for certain severe adverse pregnancy outcomes associated with low weight but increase the number at risk of macrosomia and maternal hypertension. Further research could determine whether weight‐based ART treatment strategies improve maternal and child health.

## 1 INTRODUCTION

Multiple studies have reported that tenofovir alafenamide (TAF) and dolutegravir (DTG) are associated with clinically significant weight gain, with risk of treatment‐emergent obesity (body mass index, or BMI, >30) more than three times higher after 96 weeks among women randomized to TAF/emtricitabine (FTC)/DTG compared with tenofovir disoproxil fumarate (TDF)/FTC/efavirenz (EFV) in the ADVANCE trial [1, 2]. Female gender has consistently been an independent predictor of excess weight gain on integrase inhibitor‐based antiretroviral treatment (ART) in both low‐resource [1, 2] and high‐resource [3, 4, 5] settings. In pregnancy, excess weight leads to maternal complications during labour, and adverse foetal outcomes due to gestational diabetes, hypertension and infant macrosomia. Thus, ART‐associated excess weight gain has the potential to substantially worsen maternal and infant health outcomes [6, 7, 8, 9].

However, it is also possible that increases in weight may provide benefits to pregnant women with HIV. Women on ART are known to be at increased risk of adverse birth outcomes, including preterm delivery and delivering infants who are small for gestational age (SGA), which may be related to low maternal weight and low gestational weight gain (GWG) [10, 11, 12, 13, 14, 15, 16, 17, 18, 19]. A recent study found that women starting DTG‐based ART gained significantly more weight in pregnancy than women starting EFV‐based ART, but both gained less than demographically similar women without HIV [20]. Additionally, the VESTED trial, found those randomized to start TAF/FTC/DTG in pregnancy had more GWG, but 8% fewer adverse birth outcomes, compared to women randomized to TDF/FTC/EFV [21].

Determining how ART‐associated weight gain will impact outcomes is challenging because most data on weight and pregnancy outcomes comes from resource‐rich settings with low HIV prevalence [22, 23, 24] or from lower resourced settings before ART was available [25, 26]. In the pre‐ART era, wasting and malnutrition associated with AIDS led to adverse pregnancy outcomes such as foetal growth restriction and foetal death [25, 26]. However, little is known about the effect of weight in pregnant women on ART, particularly those starting ART prior to conception with high CD4 count and viral suppression, who now represent the majority of pregnant women living with HIV (WLWHIV) [27]. Also, pre‐pregnancy weight may impact pregnancy outcomes more than GWG [23, 24], so ART‐associated weight gain is likely to have the greatest impact among women on ART prior to pregnancy.

To better understand how ART‐associated increases in weight over time will impact pregnancy we first need data on the impact of maternal weight on pregnancy outcomes among WLWHIV. We utilized the Tsepamo study in Botswana to define the existing associations between baseline weight and weight gain on pregnancy outcomes among women receiving ART from conception. We also evaluated differences in these associations by ART regimen (TDF/XTC/DTG or TDF/XTC/EFV, where XTC indicates either emtricitabine [FTC] or lamivudine [3TC]).

## 2 METHODS

### 2.1 Study population and setting

This analysis included women documented to be on ART at conception with singleton pregnancies who delivered 15 August 2014 to 30 April 2020 in the Tsepamo study in Botswana. All women had to have a weight measurement during antenatal care before 24 weeks so that the weight measurement occurred before the birth outcomes, which start at ≥24 weeks’ gestational age (GA). Pregnancies ending <24 weeks are considered miscarriage and are not captured in Tsepamo. Methodologic details of Tsepamo have been previously described [10, 11]. Briefly, research assistants abstract data from the obstetric record (a record of antenatal care and delivery) at the time of delivery from all women delivering at study sites. From the obstetric record, we recorded maternal demographics, medical history, medications prescribed during pregnancy, diagnoses, clinical visits and hospital admissions during pregnancy, maternal HIV status, dates of HIV diagnosis and ART initiation and ART treatment regimens. Infant GA, vital status, birthweight and gender were also recorded for each delivery. GA was calculated at the time of delivery by the midwife using the estimated date of delivery determined during antenatal care, typically using the reported last menstrual period (LMP). Every maternal weight and blood pressure measured during an antenatal clinic appointment was recorded (with the date). Blood pressures were single measurements and measurements during labour in the inpatient setting were not collected. Pre‐pregnancy weight was by maternal self‐report. Data on height were not available, preventing calculation of BMI.

The national HIV treatment programme in Botswana provides free testing, medical care and ART treatment. During the study period, TDF/FTC/EFV was the first‐line recommended regimen for ART initiation among ART‐naïve individuals until May 2016, when it was replaced by TDF/FTC/DTG, which was subsequently replaced by TDF/3TC/DTG in September 2018. Patients stable on previously recommended regimens were not switched to the newer regimens (with the exception of the TDF/FTC/DTG formulation being replaced by TDF/3TC/DTG), so there were many women on ART with nevirapine (NVP), lopinavir‐ritonavir (LPV‐r) and EFV who delivered throughout the study.

### 2.2 Exposures

Baseline weight was defined as the initial weight in kilograms (kgs) measured during pregnancy at <24 weeks’ GA, categorized in 10 kg increments (<50, 50 to 60, 60 to 70, 70 to 80, 80 to 90 and >90 kg). We felt this definition would be more accurate than using pre‐pregnancy weight which was self‐reported, and less biased than restricting to only first trimester measured weight in pregnancy, which was available for a smaller proportion of women who may have presented to antenatal care early due to complications/comorbidities. GWG was defined as the average weekly weight gain in the second trimester, using the difference in weight at 12 ± 2 and 24 ± 2 weeks, divided by the number of weeks between measurements (kg/week). Limiting GWG to the second trimester was necessary so that the exposure (weight gain) comes before the birth outcome (which occurs ≥24 weeks’ GA). GWG was categorized (<0.15, 0.15 to 0.25, 0.25 to 0.35, 0.35 to 0.45, 0.45 to 0.55 and >0.55 kg/week) based on the distribution of weight gain in the Botswana population, and closely approximate Institute of Medicine (IOM) recommendations for inappropriately low (0.18 kg/week) and inappropriate high (0.59 kg/week) second and third trimester weight gain (though IOM guidelines are based on pre‐pregnancy BMI) [20, 22]. For both baseline weight and second trimester weight gain, the strata that included the median baseline weight/weight gain was defined as the referent (moderate) strata. Weight strata below the referent were considered low baseline weight/low weight gain while strata above the referent were considered high baseline weight/high weight gain.

### 2.3 Outcomes

Severe birth outcomes considered in our analysis were very preterm delivery (<32 weeks’ GA), very SGA (<3rd percentile weight‐for‐GA per INTERGROWTH‐21 norms), or perinatal death (stillborn or in‐hospital neonatal death at <28 days old). “Any severe birth outcome” refers to a composite endpoint of very preterm, very SGA and/or perinatal death. Macrosomia was defined as birthweight >4000 g. The primary definition of hypertension in pregnancy was any elevated systolic blood pressure (SBP) >140 or diastolic blood pressure (DBP) >90 during pregnancy.

### 2.4 Covariates

Maternal age, occupation, marital status, education and parity were self‐reported at the first antenatal clinic visit. CD4 count testing is recommended every 6 to 12 months for people with stable HIV in Botswana, and was recorded in Tsepamo if obtained during pregnancy. Duration of ART prior to pregnancy was calculated from the start date of ART to the estimated date of conception (<1, 1 to 2, 2 to 3, >3 years). Site of delivery was defined as tertiary (2 sites) versus non‐tertiary (all other sites).

### 2.5 Statistical analysis

For the primary analysis, we described the population distribution of baseline weight and second trimester weight gain and calculated the prevalence of each individual outcome by (1) baseline weight, (2) second trimester weight gain and (3) second trimester weight gain stratified by baseline weight. The association of baseline weight and second trimester weight gain with each outcome was evaluated separately using multivariable log binomial regression to estimate relative risk (RR). Variables considered potential confounders (a‐priori) were evaluated individually, and included in adjusted models if they changed the effect estimate by >5%. We included evaluation of baseline weight as a potential confounder in the relationship between second trimester weight gain and outcomes. However, we hypothesized that second trimester weight gain was a mediator between baseline weight and outcomes, and did not adjust for weight gain in models of baseline weight and outcomes, as we did not want to condition on a mediator.

A secondary analysis was performed to test for interaction between baseline weight and outcomes by ART regimen. Given our primary interest in understanding how pregnancy outcomes may be impacted by the increasing use of DTG (which is replacing EFV in most HIV treatment programmes globally) [28, 29] we chose to limit this analysis to TDF/XTC/DTG versus TDF/XTC/EFV. We fit adjusted log binomial models as described above to evaluate each individual outcome by baseline weight, and included ART regimen (DTG/EFV) and an interaction term (for DTG/EFV).

Several sensitivity analyses were also performed, including (1) defining baseline weight as pre‐pregnancy weight, (2) defining baseline weight as measured weight <14 weeks’ GA and (3) limiting the analysis to women with a known CD4 count. The latter analysis was considered a sensitivity analysis because less than 30% of the study population had a reported CD4, and less than 3% of women in our study population had CD4 count <200, so we expected minimal confounding bias via increased risk for infections.

### 2.6 Ethics

Ethical approval for this study, including waiver of informed consent, was granted by Human Research and Development Council in Botswana and by the IRB at the Harvard T.H. Chan School of Public Health.

## 3 RESULTS

**Figure 1:**
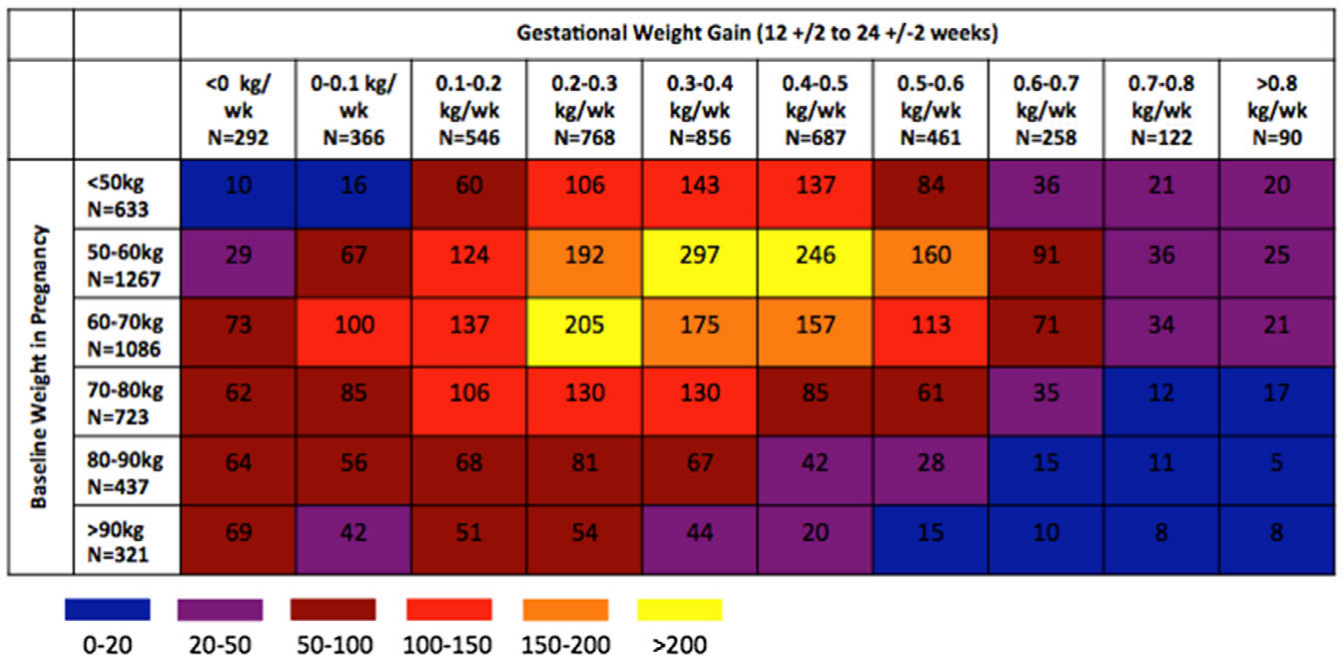
Heatmap showing the distribution of baseline pregnancy weight and second trimester weight gain among women on ART at conception in Botswana. Only women with a known baseline weight and a known second trimester weight gain are included (N = 4437). Each cell represents the number of women within that individual cell. For example, there were 10 women with baseline weight of <50 kg who also had ≤0 kg/week second trimester weight gain.

A total of 22,828 women on ART at conception delivered a singleton infant between 15 August 2015 and 30 April 2020, of whom 21,406 (94%) had a weight measured during pregnancy. The analysis of baseline weight includes 16,300 (76%) who had a weight measurement before 24 weeks’ gestation. Of these women, 4437 had weight measurements available at both 12 (±2) weeks and 24 (±2) weeks and were included in the analysis of second trimester weight gain. The median baseline weight was 63.0 kg [interquartile range (IQR) 54.4, 74.0], 13% of women had very low baseline weight (<50 kg) and 7% had very high baseline weight (>90 kg). The mean second trimester weight gain was 0.33 kg/week, 21% had very low second trimester weight gain (<0.15 kg/week) and 15% had very high second trimester weight gain (>0.55 kg/week). Low baseline weight was associated with younger age, lower educational attainment, lack of employment, nulliparity and slightly lower median CD4 count (Table 1). Low second trimester weight gain was associated with lower educational attainment, lack of employment, grand multiparity (>5 prior pregnancies) and slightly higher median CD4 count (Table 2). Women with lower baseline weight tended to have moderate to high second trimester weight gain while women with higher baseline weight tended to have lower second trimester weight gain (Figure 1).

**Table 1:** Characteristics of women on ART at conception by baseline weight in pregnancy (<24 weeks’ gestational age)

	Baseline weight (kg)					
	Low		Moderate		High	
	<50 (N = 2062)	50 to 60 (N = 4587)	60 to 70 (N = 4244)	70 to 80 (N = 2692)	80 to 90 (N = 1559)	>90 (N = 1156)
Gestational weeks at measured weight, median [IQR]	15 [12, 18]	16 [12, 19]	16 [13, 20]	16 [12, 19]	16 [12, 19]	16 [12, 19]
Maternal Age, median [IQR] years	29 [24, 34]	31 [26, 35]	33 [29, 37]	34 [30, 38]	35 [31, 38]	35 [31, 38]
No/primary school education	313 (15%)	590 (13%)	506 (12%)	294 (11%)	169 (11%)	112 (9.8%)
Current student	44 (2.2%)	112 (2.5%)	58 (1.4%)	28 (1.1%)	9 (0.6%)	8 (0.7%)
Unemployed, No. (%)	1359 (68%)	2751 (62%)	2289 (56%)	1339 (51%)	735 (49%)	517 (46%)
Married, No (%)	93 (4.6%)	346 (7.7%)	500 (12%)	465 (18%)	294 (19%)	239 (21%)
Nulliparity, No. (%)	436 (21%)	656 (14%)	377 (8.9%)	121 (4.5%)	63 (4.0%)	37 (3.2%)
≥5 prior pregnancies	338 (16%)	967 (21%)	1055 (25%)	722 (27%)	436 (28%)	355 (31%)
Number of antenatal care visits, median [IQR]	10 [8, 12]	10 [8, 12]	10 [8, 13]	11 [8, 13]	11 [9, 13]	11 [9, 13]
Delivery at tertiary care hospital No. (%)	814 (40%)	1857 (41%)	1700 (40%)	1144 (43%)	664 (43%)	530 (46%)
CD4 count, median [IQR]	537.5 [414, 698]	539 [406, 673]	526 [403, 678]	562.5 [423, 721]	573 [437, 729.5]	625 [465.5, 793]
CD4 < 200 cells/mm^3^	22 (4.0%)	38 (3.3%)	23 (2.2%)	21 (3.0%)	10 (2.5%)	8 (2.4%)
CD4 > 500 cells/mm^3^	325 (59%)	683 (59%)	589 (55%)	589 (55%)	251 (63%)	232 (71%)
ART regimen contains						
DTG	374 (18%)	670 (14%)	590 (14%)	381 (14%)	231 (15%)	204 (18%)
EFV	972 (47%)	2197 (48%)	1906 (45%)	1158 (43%)	708 (46%)	518 (45%)
NVP	677 (33%)	1150 (25%)	1252 (30%)	855 (32%)	452 (29%)	309 (27%)
LPV‐r	127 (6.2%)	243 (5.3%)	205 (7.7%)	131 (4.9%)	72 (4.6%)	63 (5.5%)
Other/unknown	141 (6.9%)	289 (6.4%)	246 (5.9%)	141 (5.3%)	88 (5.7%)	51 (4.5%)

ART, antiretroviral treatment; DTG, dolutegravir; EFV, efavirenz; IQR, inter quartile range; KG, kilogram; LPV‐r, lopinavir/ritonavir; NVP, nevirapine.

**Table 2:** Characteristics of women on ART at conception by average weekly second trimester weight gain (12 ± 2 to 24 ±2 weeks’ gestational age)

	Second trimester weight gain (kg/week)					
	Low		Moderate		High	
	<0.15 (N = 906)	0.15 to 0.25 (N = 671)	0.25 to 0.35 (N = 829)	0.35 to 0.45 (N = 774)	0.45 to 0.55 (N = 603)	>0.55 (N = 654)
Gestational weeks at measured weight, median [IQR]	12 [10, 13]	12 [9, 13]	12 [10, 13]	12 [9, 13]	12 [9, 13]	12 [9, 13]
Maternal Age, median [IQR] years	33 [29, 37]	33 [28, 37]	33 [28, 36]	33 [28, 36]	32 [28, 36]	31 [27, 36]
Baseline Weight, median [IQR] kg	71.2 [60.5, 83.4]	66.0 [56.0, 76.0]	60.7 [52.6, 72.0]	59.1 [52., 69.8]	57.8 [51.5, 68.8]	60.2 [52.7, 70.0]
No/primary school education	109 (12%)	68 (10%)	68 (8%)	68 (9%)	55 (9%)	55 (8%)
Current Student	10 (1.1%)	8 (1.2%)	14 (1.7%)	16 (2.1%)	11 (1.9%)	14 (2.2%)
Unemployed, No. (%)	485 (55%)	325 (50%)	409 (51%)	393 (52%)	283 (49%)	304 (48%)
Married, No (%)	117 (13%)	105 (16%)	135 (17%)	103 (14%)	89 (15%)	76 (12%)
Nulliparity, No. (%)	77 (8.5%)	81 (12%)	104 (13%)	105 (14%)	89 (15%)	100 (15%)
≥5 prior pregnancies	196 (22%)	19 (18%)	157 (19%)	135 (17%)	103 (17%)	102 (16%)
Number of antenatal care visits, median [IQR]	12 [10, 14]	12 [10, 14]	12 [10, 14]	12 [10, 14]	12 [10, 14]	12 [10, 14]
Delivery at tertiary care hospital No. (%)	356 (39%)	278 (41%)	368 (44%)	347 (45%)	275 (46%)	290 (44%)
CD4 count, median [IQR]	555 [413, 713]	547 [395, 685]	526 [403, 678]	548 [430, 704]	490 [388, 658]	514 [399, 683]
CD4 < 200 cells/mm^3^	5 (2.0%)	7 (3.9%)	3 (1.4%)	5 (2.3%)	8 (4.9%)	8 (4.4%)
CD4 > 500 cells/mm^3^	148 (60%)	104 (58%)	133 (62%)	132 (61%)	80 (48%)	97 (53%)
ART regimen contains						
DTG	141 (16%)	98 (15%)	131 (16%)	148 (19%)	96 (16%)	107 (16%)
EFV	405 (45%)	325 (49%)	365 (44%)	336 (43%)	252 (42%)	293 (45%)
NVP	243 (27%)	172 (26%)	247 (30%)	211 (27%)	179 (30%)	182 (28%)
LPV‐r	44 (4.9%)	40 (6.0%)	42 (5.1%)	33 (4.3%)	34 (5.7%)	29 (4.5%)
Other/unknown	61 (6.8%)	33 (4.9%)	39 (4.7%)	44 (5.7%)	37 (6.2%)	38 (5.9%)

ART, antiretroviral treatment; DTG, dolutegravir; EFV, efavirenz; IQR, inter quartile range; KG, kilogram; LPV‐r, lopinavir/ritonavir; NVP, nevirapine.

### 3.1 Baseline weight and outcomes

Table 3 shows the prevalence of each outcome by baseline weight category. Very preterm delivery and very SGA decreased linearly with increasing baseline weight, and both were highest among women with baseline weight <50 kg and lowest among women with baseline weight >90 kg (5.33% vs. 2.94% very preterm and 14.72% vs. 5.75% very SGA). Perinatal death prevalence was similar across all baseline weight strata. The prevalence of macrosomia increased with increasing baseline weight, from 0.19% for baseline weight <50 kg to 6.32% for >90 kg. Maternal hypertension (median onset of 31 weeks’ GA [IQR 22, 36]) had the same pattern, from 11.02% for baseline weight <50 kg to 36.83% for baseline weight >90 kg.

**Table 3:** Prevalence of outcomes by baseline weight measured in pregnancy among women on ART at conception: overall and stratified by ART regimen (DTG vs. EFV)

	Baseline weight (kg)					
	<50	50 to 60	60 to 70	70 to 80	80 to 90	>90
	No. (%)	No. (%)	No. (%)	No. (%)	No. (%)	No. (%)
Any severe adverse outcome						
Total population	425 (20.6%)	673 (14.7%)	581 (13.7%)	304 (11.3%)	164 (10.5%)	115 (10.0%)
DTG/TDF/XTC	57 (17.8%)	58 (9.8%)	52 (9.96%)	30 (8.6%)	20 (9.8%)	17 (9.3%)
EFV/TDF/XTC	165 (18.1%)	256 (12.2%)	226 (12.3%)	108 (9.5%)	57 (8.3%)	38 (4.5%)
Very preterm delivery						
Total population	110 (5.3%)	210 (4.6%)	193 (4.6%)	98 (3.6%)	49 (3.1%)	34 (2.9%)
DTG/TDF/XTC	14 (4.4%)	23 (3.9%)	18 (3.5%)	10 (2.9%)	5 (2.4%)	4 (2.2%)
EFV/TDF/XTC	44 (4.8%)	87 (4.2%)	73 (4.0%)	37 (3.3%)	17 (2.5%)	13 (2.6%)
Very small for gestational age						
Total population	301 (14.7%)	436 (9.6%)	342 (8.1%)	167 (6.3%)	99 (6.4%)	66 (5.8%)
DTG/TDF/XTC	43 (13.5%)	32 (5.5%)	34 (6.6%)	17 (4.9%)	10 (5.0%)	11 (6.1%)
EFV/TDF/XTC	113 (12.5%)	161 (7.7%)	135 (7.4%)	58 (5.2%)	40 (5.9%)	22 (4.4%)
Perinatal death						
Total population	99 (4.8%)	176 (3.8%)	189 (4.5%)	118 (4.4%)	60 (3.9%)	54 (4.7%)
DTG/TDF/XTC	11 (3.4%)	14 (2.4%)	18 (3.5%)	13 (3.7%)	9 (4.4%)	10 (5.5%)
EFV/TDF/XTC	33 (3.6%)	62 (3.0%)	68 (3.7%)	36 (3.2%)	17 (2.5%)	17 (3.4%)
Macrosomia						
Total population	4 (0.2%)	40 (0.9%)	83 (2.0%)	73 (2.7%)	58 (3.7%)	73 (6.3%)
DTG/TDF/XTC	1 (0.3%)	9 (1.5%)	14 (2.7%)	14 (4.0%)	10 (4.9%)	11 (6.0%)
EFV/TDF/XTC	2 (0.2%)	27 (1.3%)	39 (2.1%)	36 (3.2%)	29 (4.2%)	32 (6.4%)
Maternal hypertension						
Total population	227 (11.0%)	606 (13.2%)	814 (19.2%)	648 (24.1%)	472 (30.3%)	425 (36.8%)
DTG/TDF/XTC	35 (10.9%)	78 (13.2%)	101 (19.4%)	92 (26.4%)	70 (34.2%)	70 (38.5%)
EFV/TDF/XTC	73 (8.0%)	209 (10.0%)	262 (14.2%)	205 (18.1%)	174 (25.3%)	162 (32.2%)

**Figure 2:**
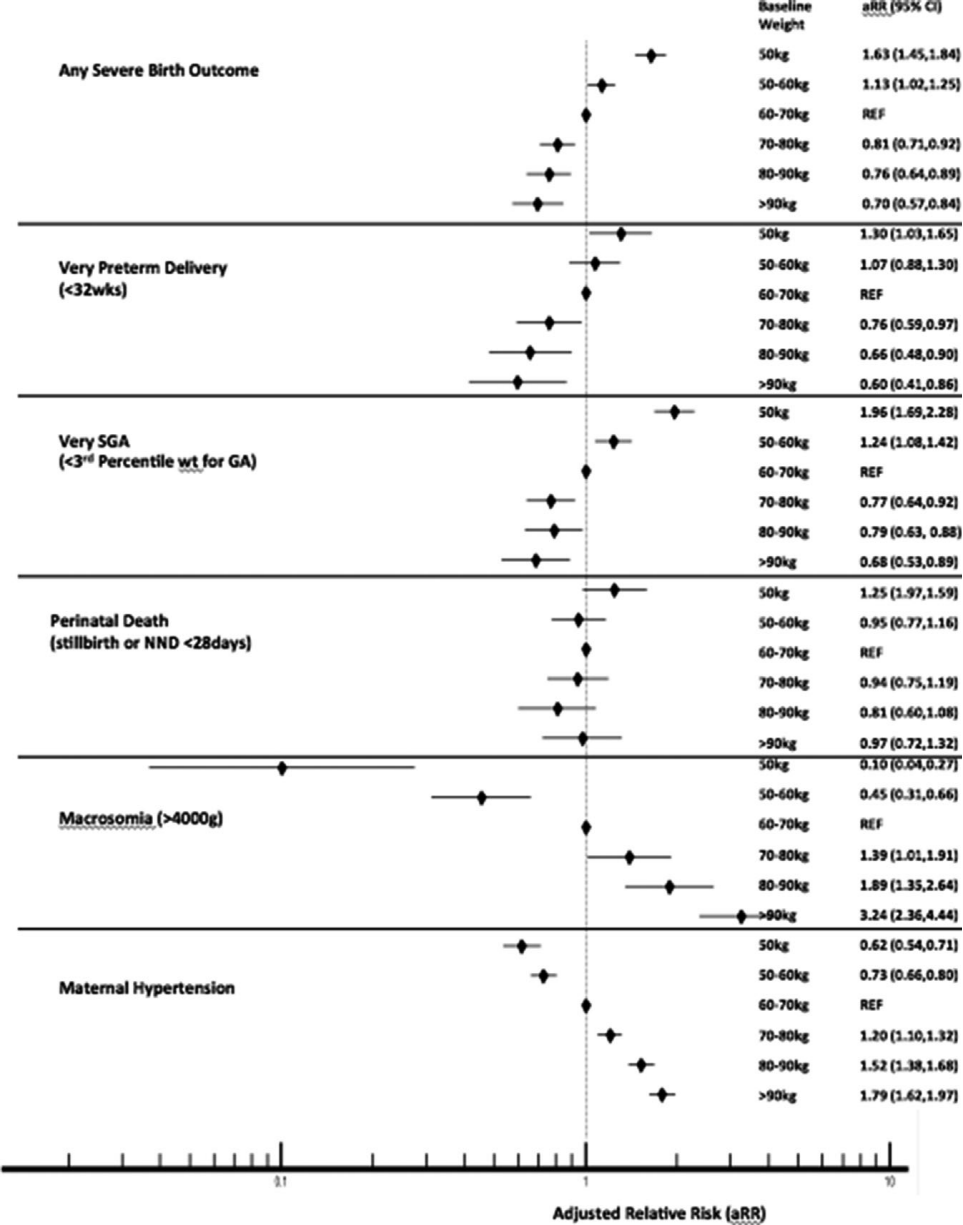
Risk of outcomes by baseline weight in pregnancy (<24 weeks GA) among women on ART at conception.

In adjusted analysis (Figure 2), compared to women with moderate baseline weight (60 to 70 kg), there was an increased risk of “any severe adverse birth outcome” and very SGA in those with lower baseline weight (<50 and 50 to 60 kg) and increased risk of very preterm delivery when baseline maternal weight was <50 kg. In contrast, there was a decreased risk of “any severe adverse birth outcome,” very SGA and very preterm delivery in women with higher baseline weight (70 to 80, 80 to 90 and >90 kg) compared with moderate baseline weight (60 to 70 kg). For the outcome of macrosomia and maternal hypertension women with low baseline weight (<50 and 50 to 60 kg) were at lower risk than women of moderate baseline weight (60 to 70 kg) while women with high baseline weight (70 to 80, 80 to 90, >90 kg) were at higher risk.

Sensitivity analyses using pre‐pregnancy weight and measured weight <14 weeks’ GA to define baseline weight yielded similar results (Table 4). Among 4218 (25.9%) of women with documented CD4 count in pregnancy, the risk of severe adverse outcomes was higher in women with CD4 > 500 than CD4 < 500 cells/mm^3^ when baseline weight was >70 kg, but lower if baseline weight was 60 kg or less, whereas macrosomia and maternal hypertension were similar across baseline weight groups when stratified by CD4 (Table 5) and there was no significant interaction by CD4 count for any of the outcomes.

**Table 4:** Sensitivity analysis for prevalence and adjusted relative risk (aRR) of outcomes by pre‐pregnancy weight and by baseline weight at <14 weeks’ GA among women on ART at conception

	Maternal weight					
	<50 kg	50 to 60 kg	60 to 70 kg	70 to 80 kg	80 to 90 kg	>90 kg
Any severe adverse outcome						
Pre‐pregnancy weight	242 (18.5%)	324 (13.1%)	233 (13.2%)	123 (11.4%)	60 (10.2%)	33 (7.3%)
aRR (95% CI)	1.51 (1.27, 1.79)	0.99 (0.85, 1.17)	REF	0.85 (0.69, 1.04)	0.76 (0.58, 1.00)	0.53 (0.37, 0.76)
Baseline Wt < 14 weeks’ GA	210 (20.2%)	272 (13.4%)	206 (11.8%)	126 (10.8%)	69 (10.3%)	40 (7.7%)
aRR (95% CI)	1.86 (1.55, 2.24)	1.20 (1.01, 1.43)	REF	0.88 (0.71, 1.09)	0.86 (0.66, 1.11)	0.64 (0.46, 0.89)
Very preterm delivery						
Pre‐pregnancy weight	65 (5.0%)	109 (4.4%)	64 (3.6%)	34 (3.2%)	20 (3.4%)	13 (2.9%)
aRR (95% CI)	1.48 (1.04, 2.09)	1.19 (0.87, 1.62)	REF	0.86 (0.57, 1.30)	0.92 (0.56, 1.52)	0.79 (0.44, 1.43)
Baseline Wt < 14 weeks’ GA	55 (5.3%)	87 (4.3%)	67 (3.8%)	41 (3.5%)	17 (2.5%)	8 (1.6%)
aRR (95% CI)	1.51 (1.05, 2.17)	1.20 (0.87, 1.65)	REF	0.86 (0.59, 1.28)	0.58 (0.33, 1.01)	0.35 (0.16, 0.76)
Very small for gestational age						
Pre‐pregnancy weight	166 (12.8%)	202 (8.2%)	134 (7.7%)	75 (7.0%)	34 (5.8%)	15 (3.4%)
aRR (95% CI)	1.81 (1.45, 2.26)	1.08 (0.98, 1.34)	REF	0.90 (0.69, 1.19)	0.75 (0.52, 1.08)	0.41 (0.24, 0.70)
Baseline Wt < 14 weeks’ GA	148 (14.3%)	177 (8.8%)	117 (6.8%)	72 (6.2%)	43 (6.5%)	26 (5.1%)
aRR (95% CI)	2.34 (1.84, 2.97)	1.37 (1.09, 1.73)	REF	0.92 (0.69, 1.23)	0.95 (0.67, 1.34)	0.76 (0.50, 1.15)
Perinatal death						
Pre‐pregnancy weight	55 (4.2%)	92 (3.7%)	84 (4.8%)	39 (3.6%)	23 (3.9%)	18 (4.0%)
aRR (95% CI)	1.00 (0.71, 1.41)	0.80 (0.59, 1.07)	REF	0.73 (0.50, 1.07)	0.80 (0.51, 1.26)	0.82 (0.50, 1.36)
Baseline Wt < 14 weeks’ GA	51 (4.9%)	76 (3.7%)	80 (4.6%)	52 (4.4%)	25 (3.7%)	16 (3.1%)
aRR (95% CI)	1.20 (0.84, 1.72)	0.89 (0.65, 1.22)	REF	0.93 (0.65, 1.32)	0.76 (0.48, 1.21)	0.64 (0.37, 1.10)
Macrosomia						
Pre‐pregnancy Weight	11 (0.8%)	32 (1.3%)	35 (2.0%)	35 (3.3%)	17 (2.9%)	36 (8.0%)
aRR (95% CI)	0.43 (0.22, 0.85)	0.66 (0.41, 1.06)	REF	1.70 (1.07, 2.71)	1.50 (0.84, 2.67)	4.11 (2.58, 6.53)
Baseline Wt < 14 weeks’ GA	3 (0.3%)	20 (1.0%)	36 (2.1%)	42 (3.6%)	31 (4.6%)	29 (5.6%)
aRR (95% CI)	0.15 (0.05, 0.50)	0.51 (0.30, 0.89)	REF	1.79 (1.14, 2.80)	2.27 (1.40, 3.69)	2.75 (1.68, 4.50)
Maternal hypertension						
Pre‐pregnancy weight	124 (9.6%)	394 (16.0%)	326 (18.6%)	297 (27.7%)	179 (30.3%)	159 (35.2%)
aRR (95% CI)	0.58 (0.48, 0.71)	0.89 (0.78, 1.02)	REF	1.45 (1.26, 1.67)	1.56 (1.34, 1.83)	1.73 (1.47, 2.03)
Baseline Wt < 14 weeks’ GA	120 (11.6%)	294 (14.5%)	358 (20.6%)	312 (26.6%)	220 (32.8%)	208 (40.2%)
aRR (95% CI)	0.60 (0.49, 0.73)	0.74 (0.64, 0.85)	REF	1.24 (1.09, 1.42)	1.56 (1.35, 1.80)	1.88 (1.6, 2.2)

**Table 5:** Sensitivity analysis of baseline maternal weight (<24 weeks) and outcomes, stratified by CD4 count

	Baseline weight (kg)					
	<50	50 to 60	60 to 70	70 to 80	80 to 90	>90
	No. (%)	No. (%)	No. (%)	No. (%)	No. (%)	No. (%)
Any severe adverse outcome						
CD4 < 500	40 (17.8%)	64 (13.3%)	63 (13.1%)	24 (8.7%)	12 (5.6%)	11 (5.1%)
aRR (95% CI)	1.46 (1.01, 2.11)	1.01 (0.72, 1.42)	REF	0.64 (0.41, 1.01)	0.62 (0.35, 1.13)	0.85 (0.47, 1.56)
CD4 > 500	51 (15.7%)	84 (12.3%)	79 (13.4%)	50 (11.6%)	26 (10.4%)	22 (9.5%)
aRR (95% CI)	1.31 (0.93, 1.83)	1.00 (0.74, 1.34)	REF	0.84 (0.60, 1.18)	0.79 (0.52, 1.20)	0.69 (0.44, 1.09)
Very preterm delivery						
CD4 < 500	12 (5.3%)	23 (4.8%)	15 (3.1%)	5 (1.8%)	1 (0.67%)	1 (1.0%)
aRR (95% CI)	1.9 (0.9, 4.1)	1.7 (0.9, 3.2)	REF	0.61 (0.22, 1.7)	0.23 (0.03, 1.72)	0.34 (0.05, 2.57)
CD4 > 500	7 (2.2%)	17 (2.5%)	27 (4.6%)	13 (3.0%)	4 (1.59%)	4 (1.72%)
aRR (95% CI)	0.54 (0.23, 1.24)	0.57 (0.32, 1.05)	REF	0.62 (0.32, 1.19)	0.32 (0.11, 0.91)	0.26 (0.08, 0.86)
Very small for gestational age						
CD4 < 500	28 (12.5%)	39 (8.2%)	43 (9.0%)	15 (5.5%)	8 (5.4%)	6 (6.3%)
aRR (95% CI)	1.51 (0.95, 2.39)	0.88 (0.57, 1.36)	REF	0.62 (0.35, 1.11)	0.62 (0.29, 1.29)	0.69 (0.30, 1.59)
CD4 > 500	42 (13.0%)	63 (9.3%)	45 (7.7%)	29 (6.8%)	16 (6.4%)	14 (6.1%)
aRR (95% CI)	1.81 (1.19, 2.75)	1.29 (0.89, 1.89)	REF	0.86 (0.54, 1.37)	0.86 (0.49, 1.51)	0.84 (0.47, 1.50)
Perinatal death						
CD4 < 500	10 (4.4%)	17 (3.5%)	21 (4.4%)	12 (4.4%)	4 (2.7%)	6 (6.3%)
aRR (95% CI)	1.16 (0.55, 2.46)	0.89 (0.47, 1.69)	REF	0.92 (0.45, 1.90)	0.63 (0.22, 1.82)	1.40 (0.58, 3.41)
CD4 > 500	10 (3.1%)	17 (2.5%)	26 (4.4%)	18 (4.2%)	8 (3.2%)	8 (3.5%)
aRR (95% CI)	0.91 (0.44, 1.90)	0.66 (0.36, 1.22)	REF	0.90 (0.49, 1.66)	0.71 (0.32, 1.57)	0.68 (0.30, 1.57)
Macrosomia						
CD4 < 500	0 (0%)	5 (1.0%)	6 (1.3%)	8 (2.9%)	5 (3.4%)	7 (7.3%)
aRR (95% CI)	0	0.80 (0.24, 2.62)	REF	2.37 (0.83, 6.77)	2.74 (0.85, 8.88)	5.93 (2.02, 17.43)
CD4 > 500	0 (0%)	9 (1.3%)	10 (1.7%)	12 (2.8%)	11 (4.4%)	9 (3.9%)
aRR (95% CI)	0	0.77 (0.32, 1.90)	REF	1.62 (0.70, 3.71)	2.49 (1.07, 5.81)	1.99 (0.795.00)
Maternal hypertension						
CD4 < 500	26 (11.6%)	66 (13.7%)	88 (18.4%)	66 (24.0%)	49 (32.9%)	32 (33.7%)
aRR (95% CI)	0.69 (0.46, 1.05)	0.81 (0.60, 1.08)	REF	1.21 (0.91, 1.61)	1.66 (1.23, 2.23)	1.69 (1.21, 2.37)
CD4 > 500	30 (9.2%)	86 (12.6%)	109 (18.5%)	97 (22.6%)	74 (29.5%)	81 (35.1%)
aRR (95% CI)	0.49 (0.33, 0.74)	0.72 (0.55, 0.93)	REF	1.19 (0.93, 1.52)	1.56 (1.20, 2.01)	1.78 (1.39, 2.29)

### 3.2 Second trimester weight gain and outcomes

**Figure 3:**
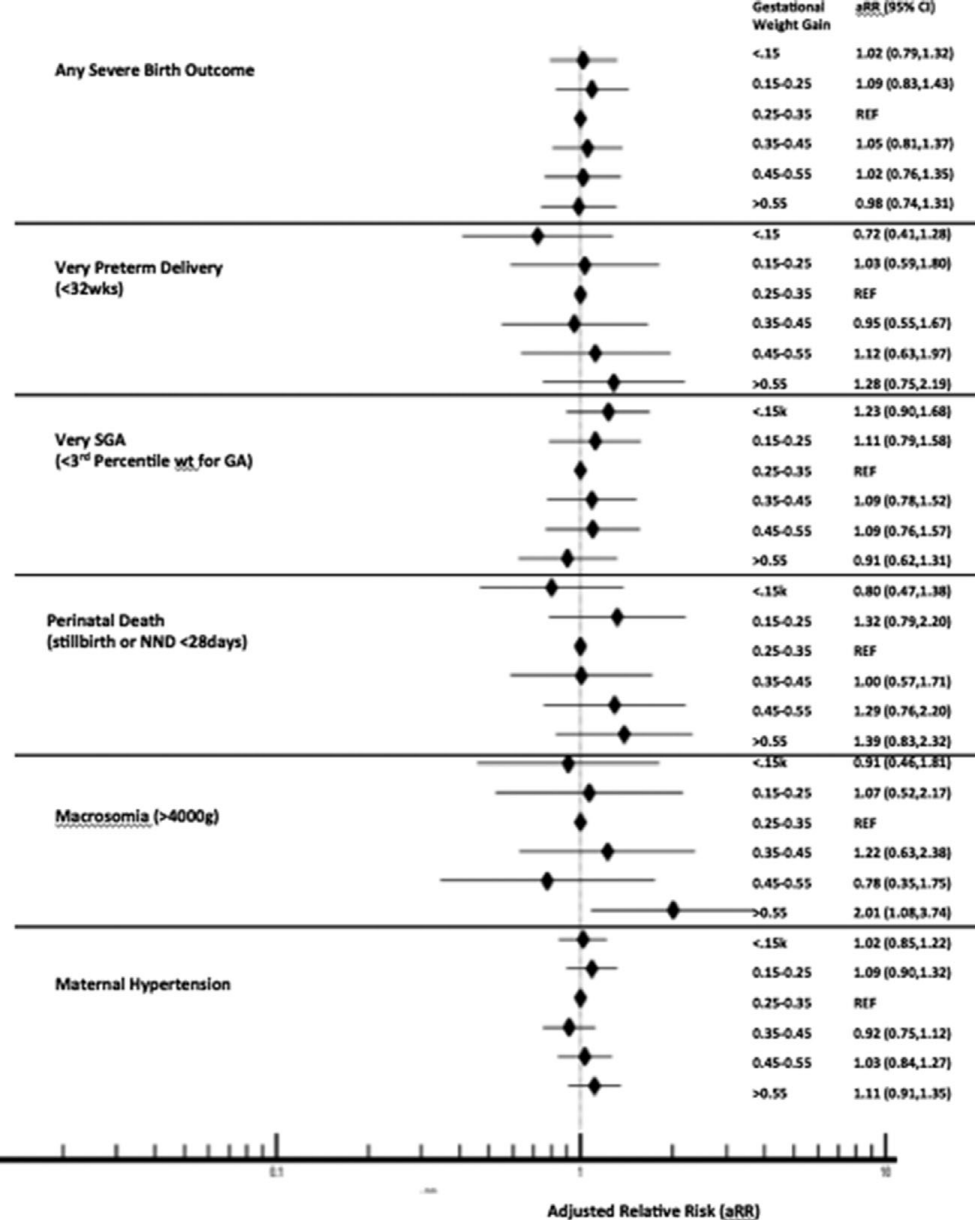
Risk of outcomes by second trimester weight gain in pregnancy (12 ± 2 weeks to 24 ± 2 week GA) among women on ART at conception.

Overall, average second trimester weight gain had a smaller effect on outcomes than baseline weight (Figure 3), and associations did not change substantially when additionally controlling for baseline weight (Table 6). Compared to moderate weight gain (0.25 to 0.35 kg/week), the prevalence of very SGA was higher and the prevalence of very preterm, perinatal death and macrosomia was in the lowest weight gain category (<0.15 kg/week), but these differences were not significant in adjusted analyses. Macrosomia, very preterm delivery and perinatal death were highest in women who gained the most weight (>0.55 kg/week), though the only significant difference in the adjusted analysis was increased risk of macrosomia (aRR 2.01, 95% CI 1.08, 3.74) when compared to 0.25 to 0.35 kg/week.

**Table 6:** Second trimester weight gain (kg/week) between 12 ± 2 and 24 ± 2 weeks’ gestational age and outcomes, with and without adjusting for baseline weight (BLW)

	Second trimester weight gain (kg/week)					
	<0.15	0.15 to 0.25	0.25 to 0.35	0.35 to 0.45	0.45 to 0.55	>0.55
Any severe adverse outcome (N, %)	113 (12.4%)	88 (13.1%)	100 (12.1%)	99 (12.8%)	71 (11.8%)	78 (11.9%)
aRR w/o BLW (95% CI)	1.02 (0.79, 1.32)	1.09 (0.83, 1.43)	REF	1.05 (0.81, 1.37)	1.02 (0.76, 1.35)	0.98 (0.74, 1.31)
aRR w/BLW (95% CI)	1.11 (0.86, 1.44)	1.14 (0.87, 1.50)	REF	1.04 (0.79, 1.34)	0.98 (0.74, 1.31)	0.99 (0.74, 1.31)
Very preterm delivery (N, %)	22 (2.4%)	22 (3.3%)	26 (3.1%)	25 (3.2%)	21 (3.5%)	28 (4.3%)
aRR w/o BLW (95% CI)	0.72 (0.41, 1.23)	1.03 (0.59, 1.80)	REF	0.95 (0.55, 1.66)	1.12 (0.63, 1.97)	1.28 (0.75, 2.20)
aRR w/BLW (95% CI)	0.76 (0.43, 1.36)	1.06 (0.61, 1.86)	REF	0.94 (0.54, 1.64)	1.09 (0.62, 1.93)	1.29 (0.75, 2.20)
Very small for gestational age (N, %)	86 (9.5%)	58 (8.7%)	65 (7.9%)	64 (8.3%)	49 (8.2%)	47 (7.2%)
aRR w/o BLW (95% CI)	1.23 (0.90, 1.68)	1.11 (0.79, 1.58)	REF	1.09 (0.78, 1.52)	1.09 (0.76, 1.56)	0.91 (0.62, 1.32)
aRR w/BLW (95% CI)	1.37 (0.997, 1.80)	1.19 (0.84, 1.68)	REF	1.07 (0.77, 1.50)	1.06 (0.74, 1.51)	0.92 (0.63, 1.33)
Perinatal death (N, %)	26 (2.9%)	30 (4.5%)	27 (3.3%)	26 (3.4%)	25 (4.1%)	31 (4.7%)
aRR w/o BLW (95% CI)	0.80 (0.47, 1.38)	1.32 (0.79, 2.20)	REF	1.00 (0.59, 1.71)	1.29 (0.76, 2.20)	1.39 (0.83, 2.32)
aRR w/BLW (95% CI)	0.82 (0.48, 1.42)	1.33 (0.75, 2.23)	REF	1.00 (0.58, 1.70)	1.28 (0.75, 2.18)	1.39 (0.83, 2.24)
Macrosomia (N, %)	16 (1.8%)	14 (2.1%)	16 (1.9%)	18 (2.3%)	10 (1.7%)	27 (4.1%)
aRR w/o BLW (95% CI)	0.91 (0.46, 1.81)	1.07 (0.52, 2.17)	REF	1.22 (0.63, 2.38)	0.78 (0.35, 1.75)	2.01 (1.08, 3.74)
aRR w/BLW (95% CI)	0.80 (0.40, 1.59)	0.99 (0.49, 2.02)	REF	1.25 (0.64, 2.43)	0.81 (0.36, 1.81)	1.94 (1.04, 3.60)
Maternal hypertension (N, %)	201 (22.2%)	153 (22.8%)	170 (20.5%)	149 (19.3%)	126 (20.9%)	149 (22.7%)
aRR w/o BLW (95% CI)	1.02 (0.85, 1.22)	1.09 (0.90, 1.32)	REF	0.92 (0.75, 1.12)	1.03 (0.84, 1.27)	1.11 (0.91, 1.35)
aRR w/BLW (95% CI)	0.96 (0.80, 1.15)	1.04 (0.86, 1.26)	REF	0.93 (0.76, 1.13)	1.06 (0.86, 1.29)	1.10 (0.91, 1.34)

**Figure 4:**
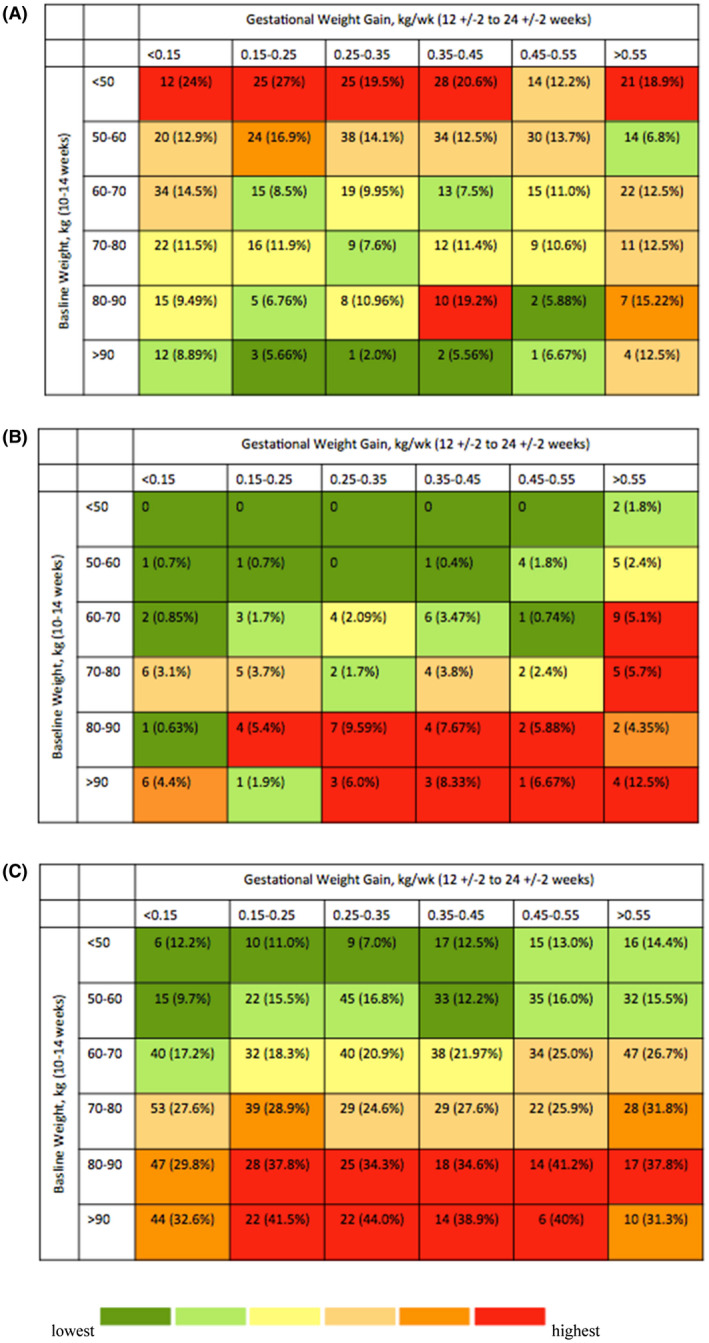
Heatmaps of baseline weight (between 10 and 14 weeks GA) and outcomes (any severe adverse outcome, macrosomia and maternal hypertension), stratified by second trimester weight gain (between 12 ± 2 and 24 ± 2 weeks GA), for **(A)** severe birth outcomes, **(B)** macrosomia, **(C)** maternal hypertension. Each cell represents the number of outcomes and the percentage of outcomes among women in that individual cell.

Figure 4 shows the prevalence of each outcome by categories of second trimester weight gain, stratified by categories of baseline weight. The highest prevalence of severe birth outcomes occurred among the women with the lowest baseline weight, regardless of second trimester weight gain, though there was also increased prevalence among women with high second trimester weight gain (>0.55 kg/week), regardless of baseline weight (Figure 4A). Macrosomia prevalence was highest among women of higher baseline weight with higher second trimester weight gain, and lowest among women with low baseline weight, particularly with lower second trimester weight gain (Figure 4B). Maternal hypertension was highest among women of higher baseline weight across strata of weight gain, except for a modest decrease in those with very low second trimester weight gain (Figure 4C).

### 3.3 Baseline weight and outcomes stratified by ART regimen

Compared with women on DTG, women on EFV were older on average, less often primigravid, and had been on ART for a longer duration prior to pregnancy (198 vs. 65 weeks), but had a similar median baseline weight (62.5 vs. 62.9 kg). Slightly more women on DTG than EFV had baseline weight <50 kg (14.8% vs. 12.7%) or >90 kg (8.4% vs. 7.0%). When restricting only to those who started ART in the time period when DTG was available (since May 2016), baseline weight was still similar (62.9 kg for DTG vs. 62.3 kg for EFV), but second trimester weight gain was greater with DTG than with EFV (0.33 vs. 0.27 kg/week). Although median baseline weight did not differ by length of time on ART prior to conception for EFV (62.0 kg <1 year, 61.5 kg 1 to 2 years, 62.0 kg 2 to 3 years; *p* = 0.37), it increased with ART duration for DTG (62.5 kg <1 year, 63.3 kg 1 to 2 years, 64.4 kg 2 to 3 years; *p* = 0.11).

The prevalence of any severe adverse birth outcome, very SGA and perinatal death was all higher in women with very high baseline weight (>90 kg) who were on DTG rather than EFV but these same outcomes were *less* common in women on DTG with baseline weight 50 to 60 and 60 to 70 kg (Table 3). However, ART regimen was not a significant effect modifier for these outcomes in adjusted analyses. The prevalence of maternal hypertension was higher among women on DTG compared with EFV across all baseline weight strata, and in adjusted analyses, ART regimen was a significant effect modifier (*p* < 0.001) for the relationship between baseline weight and this outcome.

## 4 DISCUSSION

We utilized a large birth outcomes surveillance study in Botswana to conduct the largest study, to our knowledge, to evaluate the relationship between weight in pregnancy and pregnancy outcomes among women living with HIV on ART at conception, and the first in the DTG era. We found that baseline pregnancy weight had a greater impact on adverse pregnancy outcomes than second trimester weight gain. Low baseline weight increased the risk for severe birth outcomes (particularly very preterm and very SGA), whereas high baseline weight increased the risk of macrosomia and maternal hypertension.

Our findings on baseline weight and outcomes were consistent with prior studies in the general population, including studies across resource settings, and studies that defined baseline weight before or during pregnancy [23, 25, 30, 31, 32, 33, 34]. Several prior studies, primarily from the United States (US) and Europe, found a V‐shaped or U‐shaped relationship between preterm delivery and baseline maternal weight, while we found the risk of very preterm delivery decreased steadily with increasing baseline weight category [30, 34, 35]. This may be due to differences in study populations, as Botswana has less morbid obesity than the United States/Europe [36, 37] and we may have had decreased power to detect risk in the highest weight category. In addition, obesity has been specifically associated with indicated preterm delivery [38, 39], and obstetric practices may differ in Botswana such that fewer indicated preterm deliveries typically occur. The aetiology of preterm delivery may also differ in Botswana, and among WLWHIV who are on ART [10, 40].

Our study is also consistent with the results from the VESTED study [21], which is the only randomized study of a TAF/FTC/DTG regimen in pregnancy. VESTED demonstrated both higher GWG and decreased adverse birth outcomes in women randomized to TAF/FTC/DTG compared to TDF/FTC/EFV, including fewer SGA and preterm deliveries. It is noteworthy that our study also identified effect modification by ART regimen (DTG vs. EFV) in the relationship between baseline weight and hypertension, though this is of unclear significance and did not result in increased severe adverse birth outcomes with DTG. Further research is needed to understand outcome differences with additional antiretroviral combinations, and the mechanism of TAF‐ and DTG‐associated weight gain, to determine if weight‐based decisions are warranted for choosing specific ART combinations to improve pregnancy outcomes and maternal health.

A strength of our study was the large sample size with the power to evaluate multiple outcomes, including individual severe adverse birth outcomes, which have the most clinical impact. Interestingly, our data show no relationship between baseline weight and perinatal death. This may be because the increased risk of perinatal death among infants born very preterm or very SGA in low weight women was balanced by the increased risk of perinatal death with macrosomia and hypertension in high weight women. However, our results should not be interpreted as equivalency of low baseline weight with high baseline weight, as we were unable to measure many outcomes associated with weight, including gestational diabetes, post‐partum haemorrhage, infection, neonatal growth and neurodevelopment. Rather, our findings can be used to support the importance of maternal health interventions that focus on both extremes of maternal weight, particularly in high HIV‐prevalence, low resource settings, where there are currently dual epidemics of malnutrition and obesity [41].

One major limitation of our study is that we were unable to calculate BMI. Our findings may overestimate risks among women at the extremes of height and we cannot use our findings to make clinical recommendations for weight and weight gain at the individual level. While BMI is a more accurate predictor of birth outcomes than weight, it is unlikely to significantly change the interpretation of our results, as few women in the highest and lowest baseline weight categories will have a normal BMI. For example only women taller than 198.6 cm (6 feet 2.7 inches) will have a BMI < 25 with weight >90 kg, and only women shorter than 158 cm (5 feet 2 inches) have BMI > 20 with weight <50 kg. Similarly, defining baseline weight at <24 weeks’ gestation (available for 70% of women in our cohort), rather than first‐trimester (available for 31%) or pre‐pregnancy weight (available for 33%) may underestimate risk in the low weight categories and overestimate risk in the higher weight categories. However, our sensitivity analysis using baseline weight measured at <14 weeks’ GA, and reported pre‐pregnancy weight, showed that these differences were small and did not change the interpretation of results. This supports prior World Health Organization (WHO) recommendations to use weight at the first antenatal clinic visit to identify high‐risk pregnancies [34], particularly in limited resource settings, where pre‐pregnancy BMI is not routinely known.

Other limitations in our study included inadequate ability to evaluate the impact of CD4 count due to missingness; inability to account for repeat pregnancies during the study period; scales used in routine care that may not be properly calibrated; and absence of early prenatal ultrasound for GA dating. However, none of these limitations were likely to differ by weight strata. Additionally, our analysis evaluated weight gain in the second trimester, and we cannot make inferences about the effect of weight gain in the third trimester on later pregnancy outcomes. A relatively small proportion of the cohort had second trimester weight gain measured because most women do not present to antenatal care early enough to be included in this analysis, so our results may not be generalizable. Finally, blood pressure may be overestimated in obese patients if an inappropriately small cuff is used and could overestimate hypertension among women of high baseline weight in our study.

Our study results help to frame the discourse on HIV treatment optimization in women. Most of the ART‐weight gain literature has focused on risks that will increase from excess weight gain and obesity [9, 42]. However, our findings suggest there may also be benefits from weight gain if newer ART regimens lead to fewer women with low baseline weight and low weight gain in pregnancy. The balance of risks and benefits will likely vary by setting.

## 5 CONCLUSIONS

Among women on ART at conception, low baseline weight in pregnancy was associated with increased risk of severe birth outcomes while high baseline weight was associated with increased risk of macrosomia and maternal hypertension. ART‐associated weight gain with newer antiretroviral medications may have both positive and negative benefits for maternal and child health depending on the mother’s weight entering pregnancy. Further research is needed to understand how newer ART combinations differentially impact outcomes among high‐ and low‐weight women.

## Competing interest

The authors declare no conflict of interest.

## Authors’ contributions

RZ and RS designed the study, ECC, PW, RL, JH, CM, DRM, CZ, SL and BJW contributed to the analysis plan. RZ, MD, GM, JD, JuM, JoM, SL and RS performed the research. RZ conducted the statistical analysis and wrote the manuscript with significant input from RS and EC. All authors contributed to the interpretation of data and have read and approved the final manuscript.
